# PBPK-PD modeling for the preclinical development and clinical translation of tau antibodies for Alzheimer’s disease

**DOI:** 10.3389/fphar.2022.867457

**Published:** 2022-09-02

**Authors:** Peter Bloomingdale, Daniela Bumbaca-Yadav, Jonathan Sugam, Steve Grauer, Brad Smith, Svetlana Antonenko, Michael Judo, Glareh Azadi, Ka Lai Yee

**Affiliations:** ^1^ Quantitative Pharmacology and Pharmacometrics, Merck & Co., Inc., Boston, MA, United States; ^2^ ADME, Merck & Co., Inc., South San Francisco, CA, United States; ^3^ Discovery Neuroscience, Merck & Co., Inc., West Point, PA, United States; ^4^ Safety Assessment—Laboratory Animal Resources, Merck & Co., Inc., West Point, PA, United States; ^5^ Laboratory Animal Resources, Merck & Co., Inc., South San Francisco, CA, United States

**Keywords:** PBPK, QSP, antibody, tau, Alzheimer’s disease

## Abstract

Disrupted tau proteostasis and transneuronal spread is a pathological hallmark of Alzheimer’s disease. Neurodegenerative diseases remain an unmet medical need and novel disease modifying therapeutics are paramount. Our objective was to develop a mechanistic mathematical model to enhance our understanding of tau antibody pharmacokinetics and pharmacodynamics in animals and humans. A physiologically-based pharmacokinetic-pharmacodynamic (PBPK-PD) modeling approach was employed to support the preclinical development and clinical translation of therapeutic antibodies targeting tau for the treatment of Alzheimer’s disease. The pharmacokinetics of a tau antibody was evaluated in rat and non-human primate microdialysis studies. Model validation for humans was performed using publicly available clinical data for gosuranemab. In-silico analyses were performed to predict tau engagement in human brain for a range of tau antibody affinities and various dosing regimens. PBPK-PD modeling enabled a quantitative understanding for the relationship between dose, affinity, and target engagement, which supported lead candidate optimization and predictions of clinically efficacious dosing regimens.

## Introduction

Alzheimer’s disease is a central neurodegenerative disease and the leading cause of dementia. With an aging global population and the lack of effective disease modifying therapies, age-related neurodegenerative diseases are an increasing public health concern. In a collaborative effort, a systematic analysis was performed to assess the global burden of neurological disorders (Collaborators, 2019). Authors reported that the world-wide prevalence of Alzheimer’s disease (AD) was 46 million and the number of individuals suffering from AD increased by over 100% between the years of 1990–2015. The number of individuals in the US with AD is projected to increase from the current 5.8 million to 13.8 million by 2050, which will increase associated annual US healthcare costs to exceed 1 trillion USD (Alzheimer’s, 2016). Hence, there is a significant medical need for effective disease modifying therapeutics for the treatment of neurodegenerative diseases.

The complex nature of neurodegenerative disease makes it challenging to develop effective therapies. Over the last decade, there has been a considerable amount of investigation into passive immunotherapy strategies for the treatment of neurodegenerative disease. Therapeutic antibodies have been developed against proteins that aggregate under pathological conditions, such Aβ and tau, for the treatment of AD. However, the clinical success with these therapies has been limited (Panza et al., 2019). Aducanumab, an antibody against Aβ, was recently granted accelerated approval for the treatment of Alzheimer’s disease. Although aducanumab significantly decreased brain Aβ burden in two phase three clinical trials, measured via amyloid PET, improvements on clinical endpoints of cognition were only observed in one of the two trials (Knopman et al., 2021). One argument for this discrepancy is that patients in the positive trial received greater exposures of aducanumab (Schneider, 2020). This exemplifies the importance for quantitatively understanding the relationship between drug potency, exposure, and response.

There has been a rising interest in the application of mechanism-based pharmacokinetics-pharmacodynamics (PK-PD) modeling approaches, such as physiologically-based pharmacokinetic (PBPK) (Gerlowski and Jain, 1983) and quantitative systems pharmacology (QSP) (Sorger et al., 2011) modeling. Several PBPK models of the brain have recently been developed for a variety of treatment modalities, including small molecules (Saleh et al., 2021), antibodies (Bloomingdale et al., 2021), and gene therapies (Monine et al., 2021). Mechanistic modeling, in comparison to either fit-for-purpose or empirical modeling, offers more realistic representations of physiological and pathophysiological systems. Parameter values are often within biological constraints and *a priori* predictions that deviate from observed data can shed light on additional phenomena in the system that has not been mechanistically described. Hence, mechanistic models can be a useful tool for integrating and transforming data into actionable knowledge to provide guidance for drug development programs.

The objective of our research was to develop a mechanistic mathematical model to enhance our quantitative understanding for pharmacokinetics and pharmacodynamics of tau antibodies in animals and humans. To demonstrate the application of PBPK modeling to support preclinical and early clinical development, we have expanded upon a previously published brain PBPK model for antibodies to include tau protein dynamics. Tau is a protein found predominately in neurons and is responsible for the stabilization of microtubules. Under pathological conditions, tau becomes hyperphosphorylated, dissociates from microtubules, aggregates, and spreads transneuronally throughout the brain, which thought to be a primary driver of dementia (Hardy and Higgins, 1992). There are at least nine tau-targeting antibody therapeutics in clinical development for the treatment of Alzheimer’s disease, which makes this therapeutic strategy an interest across many pharmaceutical companies. Gosuranemab (BIIB092) is an N-terminal targeting tau-targeting antibody that displayed strong target engagement in the CSF of progressive supranuclear palsy (PSP) and AD patients, however it was subsequently discontinued due to a lack of efficacy (Boxer et al., 2019) (Shulman et al., 2021). The pharmacokinetics of an internally developed tau antibody was evaluated in rat and non-human primate microdialysis studies. Model validation for humans was performed using previously published clinical data for gosuranemab (Boxer et al., 2019). In-silico analyses were performed to predict tau engagement in human brain for a range of antibody affinities to tau and various dosing regimens. Using available preclinical and clinical data, our model was applied to evaluate several questions commonly faced in preclinical and early clinical development, including the design of preclinical experiments, quantitative evaluation of the benefits of affinity optimization and half-life extension, the potential impact of blood contamination in CSF samples, and clinical trial design.

## Materials and methods

### Physiologically-based pharmacokinetic-pharmacodynamic model development

A multi-species (mouse, rat, monkey, human) physiologically-based pharmacokinetic (PBPK) model for antibody therapeutics was originally developed by Shah and Betts in 2012 (Shah and Betts, 2012) and subsequently expanded by Chang et al., in 2019 (Chang et al., 2019) to include additional anatomical features and physiological processes of the brain. The Chang model consists of 100 differential equations, 15 tissues (lung, heart, kidney, muscle, skin, brain, adipose, thymus, small intestine, large intestine, spleen, pancreas, liver, bone, lymph), and a detailed brain compartment. The brain component of the PBPK model consists of vasculature, endosomal spaces of the blood-brain-barrier (BBB) and blood-cerebral-spinal-fluid-barrier (BCSFB), interstitial fluid (ISF), and four cerebral spinal fluid (CSF) compartments. A slight modification to the model was made to update plasma volume. The volume of plasma in tissue vasculature was subtracted from the total plasma volume to obtain an updated plasma volume, which was not considered in the Shah-Betts and Chang models.

The Chang model was expanded to include tau dynamics. The half-life of tau in human CSF was reported to be 30.7 days based on a stable isotope labeling kinetics (SILK) experiment (Sato et al., 2018). The intracellular turnover rate of tau has been shown to be isoform and phosphorylation status dependent using iPSC-derived neurons *in vitro* (Sato et al., 2018). However, the impact of different isoforms and post-translational modifications on the tau turnover rate *in vivo* remains unclear. Therefore, for simplicity, we have assumed that there is no difference in the extracellular elimination rate of tau versus ptau. The baseline concentration of tau in human CSF has been shown to range from 78 to 3,652 pg/ml (Herukka et al., 2015). We used a value of 1,080 pg/ml for CSF tau, which was the average concentration between the CSF tau from Herukka et al. (2015) and Sato et al. (2018). Total tau CSF concentrations on average were comparable between both of the studies. Only 3 of the 11 patients in Herukka et al. (2015) were diagnosed with AD. The 24 patients in Sato et al. (2018) ranged between cognitively normal and very mild AD (CDR scores ranged between 0–0.5). Therefore, the initial concentration of total tau in the CSF best reflects a cognitively normal to early mild AD population. The percentage of phosphorylated tau (ptau) relative to total tau in CSF is 6.79% (Herukka et al., 2015). Note that the data used in this version of the model was specifically for phosphorylated threonine 181 (pT181). Depending on the tau protein target-site of interest, the model could be adjusted to account for differences in percent phosphorylated for different phospho-epitopes. The structure of our model can be generalizable to all tau antibodies; however, the parameterization should depend on the antibody and tau target-site of interest. Using a molecular weight of 40 kDa for tau and the percentage of ptau in CSF, the concentration of ptau in human CSF was calculated to be 1.83 pM. Human brain ISF concentrations of tau were reported from patients who had a cortical brain biopsy for idiopathic normal pressure hydrocephalus (Herukka et al., 2015). The concentration of brain ISF tau is 2745.7 pg/ml, 2.54 times greater than CSF tau concentrations. The percentages of ptau to total tau in brain ISF was 10.8% (Herukka et al., 2015). Using a molecular weight of 40 kDa for tau and the percentage of ptau in brain ISF, the concentration of ptau in human brain ISF was calculated to be 7.43 pM. The tau production rate in human CSF has been reported to be 25.7 fM/h (Sato et al., 2018). Therefore, the ptau production rate in human CSF was set to 1.75 fM/h considering 6.79% of CSF tau is phosphorylated. To account for the ptau concentration difference between CSF and ISF, the tau production rate in brain ISF was assumed to be 2.54 times greater. Therefore, the ptau production rate in human brain ISF was set to 7.06 fM/h considering 10.8% of brain ISF tau is phosphorylated. The antibody tau complex was assumed to be eliminated at the same rate as an anti-tau antibody with a half-life of 28 days.

The following target binding equations were introduced into the model to describe the interaction between antibody and phosphorylated-tau (ptau).

$$\frac{dC_{{ptau}_{x}}}{dt}=k_{in\_x}-k_{out\_x}∙C_{{ptau}_{x}}-k_{on}∙C_{{ptau}_{x}}∙C_{{mAb}_{x}}+k_{off}∙C_{{mAb\_ptau}_{x}}$$ (1)

$$\frac{dC_{mAb\_{ptau}_{x}}}{dt}=k_{on}∙C_{{ptau}_{x}}∙C_{{mAb}_{x}}-k_{off}∙C_{{mAb\_ptau}_{x}}-k_{deg}∙C_{{mAb\_ptau}_{x}}$$ (2)

Where, *x* represents the concentration of ptau (C_ptau_) or antibody ptau complex (C_mAb_ptau_) in one brain ISF and four CSF compartments: lateral ventricle (LV), third-fourth ventricle (TFV), cisterna magna (CM), subarachnoid space (SAS). We have assumed no distribution of target and antibody-target complex between compartments.

### Rat microdialysis

Rat microdialysis studies were conducted by Charles River Laboratories, South San Francisco (SSF) in accordance with the Institutional Animal Care and Use Committee (IACUC) of Charles River laboratories SSF. Sixteen male Sprague Dawley rats (*n* = 5–6 per group) were group housed and provided access to food and water *ad libitum*. Animals were kept on a 12/12 h light/dark cycle with constant room temperature (22 + 2 °C) and humidity (∼50%) and acclimated for at least 7 days prior to surgery. On the day of surgery, rats were anesthetized using isoflurane (2%, 800 ml/min O_2_). Lidocaine was also used for local analgesia and carprofen for peri/post-operative analgesia. Animals were implanted with cannula into the cisterna magna and jugular vein for CSF and blood collection respectively. Animals were then implanted with a microdialysis probe (PEE membrane, CRL, the Netherlands) via stereotaxic surgery targeting the hippocampus at the following coordinates: antero-posterior = −5.3 mm to bregma, lateral = −4.8 mm to midline and ventral = −8.0 mm to dura, the tooth bar set at 0 mm. After surgery, animals were single-housed with *ad libitum* access to food and water. Approximately 24 h after surgery, brain ISF sampling was initiated for up to 28 h collection. On each ISF sampling day, microdialysis probes were connected with tubing (Peek inlet, FEP outlet) to a microperfusion pump (Harvard PHD 2000 Syringe pump, Holliston, MA or similar). Microdialysis probes were perfused with aCSF containing 147 mM NaCl, 3.0 mM KCl, 1.2 mM CaCl_2_ and 1.2 mM MgCl_2_, and 0.15% BSA at a flow rate of 0.75 μL/min. After stabilization (2 h), microdialysis samples were collected for 60-minute periods by an automated fraction collector (820 Microsampler, Univentor, Malta) into polypropylene (300 μL) mini-vials. On day 1, ISF was collected at baseline for 2 h (i.e., 4 samples). Then, rats were administered with either 10, 50, or 100 mg/kg of antibody A at a dose volume of 2 ml/kg IV and ISF collections continued for 6 h. At the end of the first ISF collection day, rats were disconnected and remained undisturbed in their home cage until the next day. On day 2, ISF was collected at timepoints 24–28 h post dosing. Following collection, all ISF samples were stored at −80C. In addition to brain ISF, serum and CSF samples were collected at baseline (∼2 h prior to treatment) (timepoints were −2, 0.5, 6, 24, and 28 h). For each serum sample, blood was collected *via* the jugular vein cannula into serum separator vials and kept at room temperature for 30 min before processing for serum (centrifugation at 4 °C, 10000 g for 5 min). Serum samples were then snap frozen on dry ice. For CSF, samples were collected via the cisterna magna cannula and snap frozen on dry ice. At the end of microdialysis experiment, rats were euthanized with CO_2_. Terminal CSF and blood were collected and snap frozen. Brains were collected and verified for probe placement.

### Monkey microdialysis

All procedures were performed in accordance with our institution’s IACUC guidelines at the Merck & Co., Inc., Rahway, NJ, United States facility, which is AAALAC-accredited (AAALAC: The Association for Assessment and Accreditation of Laboratory Animal Care International). Rhesus microdialysis studies were conducted in-house on four monkeys. Monkeys were implanted with a silicone, 5 French Cisterna Magna Port (CMP) catheter (CMC-06-SAI Infusion Technologies-Lake Vila, IL) attached to a titanium port body (Solo Port MIN-C50-Access Technologies, Skokie, IL). The catheter tip (5 mm) was surgically implanted into the cisterna magna (Gilberto et al., 2021). Implantation of CMP allows for chronic CSF collection. Following, monkeys had microdialysis cannulation of commercially available head caps (Crist Instruments). The head cap that was used had a lower profile and the monkeys adapted well to it. The head cap/cannula placement targeting the cortex utilizing the following coordinates averaged for 4 monkeys: Ear bars set to 33.25 and head cap height set to 16 followed by +21.75 mm AP to bregma and +15.5 mm ML. The skull was drilled for placement of screws to hold the headcap in place followed by a placement of grid marked on the skull. Craniotomy performed on the area marked for placement of 4 cannulas/probes. Head cap was attached to the skull using bone screws and cement. Grid was placed in head cap and 16 mm microdialysis probes/cannulas were placed in 4 slots in the grid. Dental acrylic was applied to secure the cannulas/probes to the grid. A lid was screwed to the top of the head cap to cover the probes/cannulas. Monkeys recovered 14 days and then preliminary study work was performed (brain ISF and CSF collections).

The microdialysis flow rate was set to 0.5 μL/min utilizing a Harvard CMA 402 micro syringe pump and a 2.5 ml Hamilton syringe. Microdialysis probes (CRL-PP-PE-180-040—1000kDA-manufactured by CRL-Netherlands) were perfused with Hamilton syringes containing artificial CSF (CMA-Ref P000151) and 0.15% BSA (Invitrogen-Ref 15561-020, 50 mg/ml) solution. The solution was filtered using a 0.22 μm filter (Millex GP-Ref SLGP033RB). The probes were connected to the Hamilton syringe with PEEK tubing and the collection tube (Eicom/Richell low protein binding tubes -polypropylene) on wet ice was placed ∼30 cm below the head cap. ISF collection was driven by gravity. For the study only one site was used for ISF collection. For three monkeys the same site was used for all the ISF collections days 0–21. For one monkey the same site was used for all ISF collections days 0–10 and then another site was used for days 15 and day 21 due to no patency in the original site. Collection tubes were then placed on dry ice following the 30 min collection. On study days monkeys were chaired and at 8:00 a.m. probes were inserted. A 2 h probe equilibration period was done. Dosing of antibody A at 40 mg/kg IV (cephalic vein-IV bolus over 2.25 min) was performed at 10:00 a.m. on day 0. Monkeys were in the lab on days 0 and 1 for a period of 8 h. For all study days after day 1 when serum, CSF, and brain ISF were collected, monkeys stayed in the lab for a period of 4 h. For all microdialysis sessions the first 2 h were used for probe equilibration and then the brain ISF samples were collected 2 h post-probe insert. For CSF collections the area over the CMP was prepped prior to the microdialysis probe insert utilizing 6 ml Duraprep applicator (Duraprep surgical solutions, #M Health Care). Area was allowed to dry 3 min and a single sterile Huber needle (Access Technologies) was placed and capped with a sterile injection cap. When a study time point was collected and the procedure was done under sterile conditions utilizing sterile gloves. The injection cap was removed and 0.9 ml of CSF was collected and discarded to allow for catheter volume (0.4 ml) plus an additional 0.5 ml waste. Following this a 500 μL study sample was collected and placed immediately on dry ice. The catheter was then locked using 0.4 ml sterile saline flush and the Huber needle was removed. For serum collection monkeys were bled using a butterfly and blood was taken from the saphenous veins. Blood was allowed to sit for 20 min then centrifuged at 10,000 rpm, 20°C for 5 min. A 200 and 300 μL serum sample was placed into a 1.4 ml alphanumeric tubes and immediately placed on dry ice. Feeding regimen for the study entailed on day 0, monkeys were fasted the night prior to dosing. Monkeys were given a Pedialyte 10% solution in study chairs at 2, 26, and 50 h timepoints. Monkeys were fed treats (grapes and bananas) after the 2 h, 26 h post time point in study chairs. Monkeys were fed again upon return to cages at ∼4:00 p.m. full ration on days 0 and 1. Upon return to their cages monkeys were fed a full ration. Enrichment (TV) was provided in the study room after the 4 and 28 h time points for ∼1 h.

### Bioanalysis

The concentration of Antibody A was measured by a bioanalytical method using an electrochemiluminescence based assay with a lower limit of quantitation (LLOQ) in rhesus monkey serum and CSF of 8.23 ng/ml. Briefly, 96-well flat-bottom Meso-Scale Discovery (MSD) Streptavidin Gold multi-array plates were blocked with 5% bovine serum albumin (BSA) in PBS followed by coating with biotinylated mouse anti-human Ig kappa light chain antibody in Modified ELISA Diluent buffer (MED) (0.5% BSA [wt/v], 0.05% Tween 20 [v/v], 0.25% CHAPS [wt/v], 5 mM EDTA in PBS at pH 7.4). For signal detection a sulfo-tagged mouse anti-human IgG CH2 domain antibody was used. Standards, controls, and sample dilutions followed by detection reagent were added in between sequential wash steps and incubations. Electrochemiluminescence signal proportional to captured Antibody A was captured on an MSD plate reader, Meso Sector S600. Concentrations of Antibody A were derived through comparison to a standard curve (0.41–300 ng/ml range) applying a 4 parameter non-linear regression fit. The method was qualified via assessment of accuracy, precision and dilutional linearity using spiked Antibody A samples serving as high, medium and low controls spanning the calibration curve. Assay run acceptance was determined by recovery of a minimum of 4 out 6 control samples (high, medium, low: tested in duplicates) within 20% of nominal value, as well as visual inspection of calibrator curve readings.

### Tau physiologically-based pharmacokinetic-pharmacodynamic model validation in rats, monkey, and human

Three *in vivo* preclinical experimental studies investigating the PK of Antibody A were conducted. A rat microdialysis study was performed to investigate the PK of Antibody A at single IV doses of 10, 50, and 100 mg/kg. Concentrations of Antibody A were measured in serum, CSF, and brain ISF. The PK of Antibody A was then investigated in cynomolgus monkeys at single IV doses of 3, 10, 40, and 80 mg/kg. Only serum concentrations were measured. Lastly, a microdialysis study was conducted in rhesus monkeys at a single IV dose of 40 mg/kg. Concentrations of Antibody A were measured in serum, CSF, and brain ISF. For model validation, *a priori* predictions were performed for each of these experiments and compared to observed data.

Clinical PK-PD data for BIIB092, an N-terminal targeting anti-tau antibody, was obtained from the literature and digitized. PBPK-PD model predictions were generated and overlayed with the observed clinical data of serum PK, CSF PK, and CSF N-terminal tau for three dose levels (150, 700, 2100 mg IV Q4W) over a duration of 3 months. A binding affinity of 0.131 nM was used to predict the change in free N-terminal tau in CSF, which was previously determined using an *in vitro* tau binding assay (Bright et al., 2015).

### Tau physiologically-based pharmacokinetic-pharmacodynamic model application to support antibody affinity and half-life optimization

The impact of affinity and half-life on the dynamics of tau concentrations in brain ISF was investigated using simulations from the model. Simulations were performed for four hypothetical antibodies (A1–A4) with varying levels of affinity (0.1, 0.3, 1, 3 nM). Simulations were also performed for four hypothetical antibodies (A1a–A1d) that have the same affinity (Kd = 0.1 nM) and different terminal half-lives (20, 40, 60, 80 days). For both simulation scenarios, each antibody was simulated across a range of doses from 0 to 100 mg/kg and tau occupancy at 8 weeks was calculated. Tau occupancy was calculated as follows:

$$Tau\;Occupancy\;\left(\%\right)=\frac{Bound\;Tau}{Total\;Tau}\times 100\%$$ (3)

Where bound tau is the amount of antibody tau complex, and total tau is the sum of free tau and antibody tau complex.

### Tau physiologically-based pharmacokinetic-pharmacodynamic model application to predict the impact of blood contamination on cerebral spinal fluid samples

To assess the potential impact of blood contamination on Antibody A concentration and tau dynamics in human CSF, the percent error in antibody and tau concentrations were calculated as a function of different levels of blood contamination. Blood contamination was calculated as:

$$C_{CSF\_BC}=C_{CSF}∙\left(1-F_{BC}\right)+C_{Serum}∙\left(1-HCT\right)∙F_{BC}$$ (4)

Where, C_CSF_BC_ is the concentration of Antibody A in CSF when accounting for concentration difference due to blood contamination and F_BC_ is the fraction of blood contamination. HCT is the hematocrit, represented as a fraction. C_CSF_ and C_Serum_ are the concentration of antibody in the CSF and serum, respectively. CSF tau occupancy (TO_CSF_) and CSF tau occupancy when accounting for blood contamination (TO_CSF_BC_) were calculated as:

$${TO}_{CSF}=\frac{C_{CSF}}{\left(C_{CSF}+K_{D}\right)}$$ (5)

$${TO}_{CSF\_BC}=\frac{C_{CSF\_BC}}{\left(C_{CSF\_BC}+K_{D}\right)}$$ (6)

Where, K_D_ is the antibody affinity to tau. The percent error in antibody CSF concentration (PE_C_CSF_) was calculated as:

$${PE}_{C\_CSF}=\frac{\left|C_{CSF}-C_{CSF_{BC}}\right|}{C_{CSF}}∙100\;\%$$ (7)

The precent error in CSF tau occupancy (PE_TO_CSF_) was determined using the following equation:

$${PE}_{TO\_CSF}=\frac{\left|{TO}_{CSF}-{TO}_{CSF_{BC}}\right|}{{TO}_{CSF}}∙100\;\%$$ (8)

Figures were generated for PE_C_CSF_ and PE_TO_CSF_ verses a range of F_BC_ (0–10%) to visualize the impact of blood contamination on the error of antibody concentration and target occupancy. The PBPK model was utilized to predict the concentration of antibody in CSF and serum at 1-month post-administration, which were used in the percent error calculations.

### Tau physiologically-based pharmacokinetic-pharmacodynamic model application to predict clinical exposure-response

Simulations were performed to predict the PK-PD of Antibody A in humans. Five dose levels (1, 3, 10, 30, 100 mg/kg) and three antibody affinities to tau (0.01, 0.1, 1 nM) were evaluated. The PK-PD profiles for Antibody A concentrations in serum, CSF, and brain ISF and the change in free CSF tau relative to baseline were simulated over 16 weeks.

## Results

### Tau physiologically-based pharmacokinetic-pharmacodynamic model development

**FIGURE 1:**
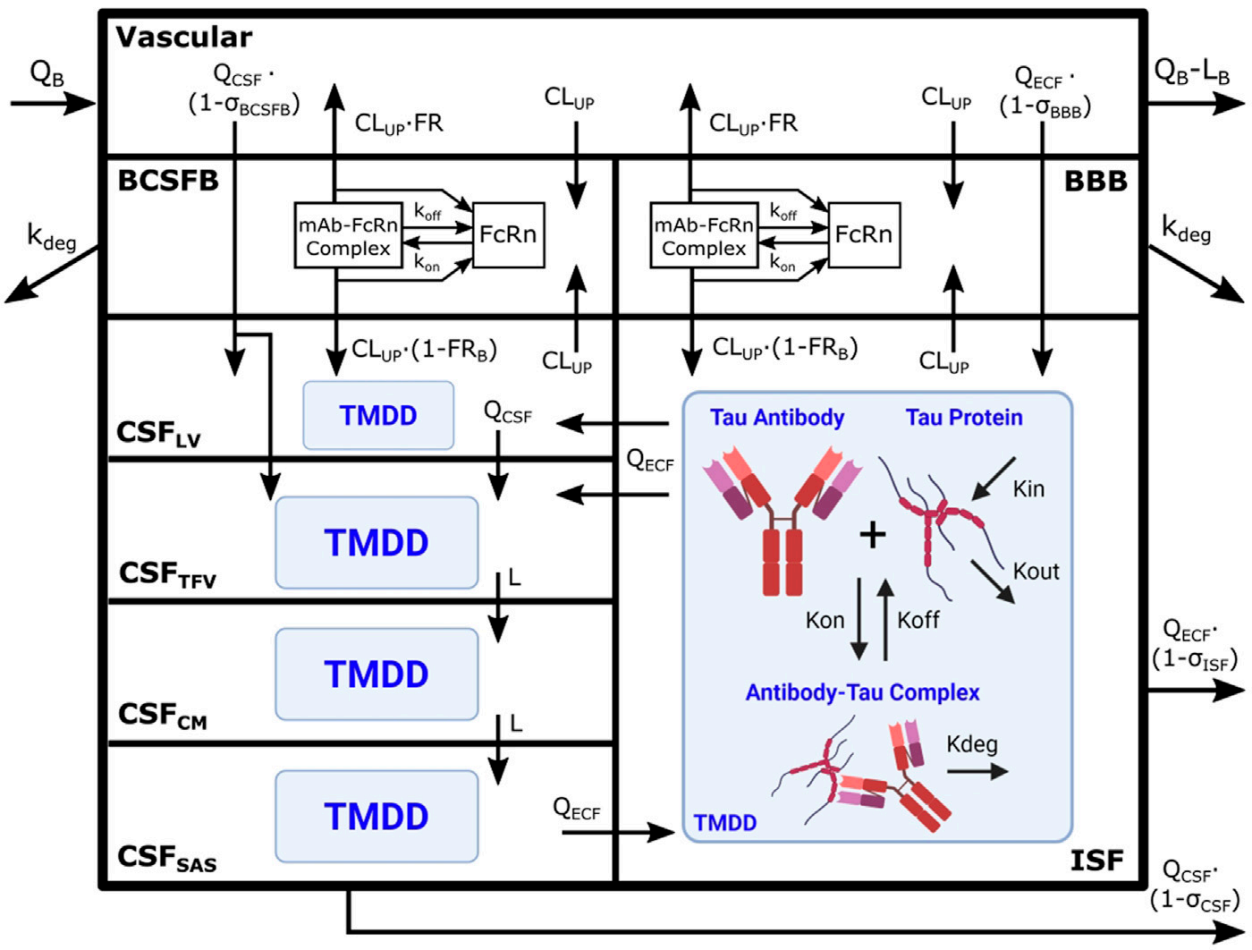
Brain PBPK model expanded to include tau dynamics. Tau antibody interactions with tau protein, or target-mediated drug disposition (TMDD), is incorporated in brain ISF and four CSF compartments. The model contains 111 differential equations and 15 tissues, but we are only depicting the brain components of the model for simplicity. Antibody enters the brain vasculature space from the plasma compartment at the brain blood flow rate (Q_B_) and leaves at a flow rate of (Q_B_–L_B_). Antibody enters the brain ISF and CSF through paracellular transport across brain barriers, BBB and BCSFB. Paracellular transport across the BBB and BCSFB is governed by brain extracellular fluid (Q_ECF_) and CSF (Q_CSF_) flow and brain vasculature reflection coefficients (σ_BBB_ and σ_BCSFB_). Antibody also enters the brain transcellularly, which is driven by non-specific pinocytosis represented in the model as an uptake clearance (CL_UP_). Antibody in the endosomal space is able to bind FcRn, form an antibody-FcRn complex, and recycle to the vasculature space or the brain. FR_B_ is the fraction of antibody that is recycled to the brain vasculature. Unbound antibody in the endosome is subjected to endosomal degradation (k_deg_). Antibody in the CSF traverses the four CSF compartments at a flow rate of Q_CSF_ or L. The four CSF compartments are lateral ventricle (LV), third-fourth ventricle (TFV), cisterna magna (CM), and subarachnoid space (SAS). Antibody is able to exchange between the brain ISF and CSF compartments via three unidirectional flows from brain ISF to CSF_LV_ and CSF_TFV_ as well as from CSF_SAS_ to brain ISF. Antibody is cleared from the brain via glymphatic clearance, which is governed by brain ISF and CSF flows (Q_ECF_ and Q_CSF_) and reflection coefficients (σ_ISF_ and σ_CSF_). Antibody in the brain ISF and CSF binds tau protein to form an antibody-tau complex, where K_on_ and K_off_ are association and dissociation rate constants. Antibody-tau complex degrades at a rate of K_deg_. Tau protein turnover is governed by production (K_in_) and elimination (K_out_) rate constants. Model diagram was created using Inkscape and BioRender.

The Chang brain PBPK model was expanded to include tau dynamics. The target-mediated effects of tau on the disposition of tau antibodies were incorporated in four CSF compartments and brain ISF (Figure 1). Model parameters for tau related dynamics are in Table 1.

**TABLE 1:** PBPK-PD model parameters for tau expression and turnover in humans.

Parameter description	Parameter	Units	Value	References
Baseline tau in CSF	Tau_0_CSF_	pM	27.0	29566794, 25720406
Baseline tau in brain ISF	Tau_0_ISF_	pM	68.6	25720406
Baseline ptau in CSF	pTau_0_CSF_	pM	1.83	29566794, 25720406
Baseline ptau in brain ISF	pTau_0_ISF_	pM	7.41	25720406
Percent ptau to total tau in CSF	—	%	6.79	25720406
Percent ptau to total tau in ISF	—	%	10.8	25720406
CSF ptau production rate	K_in_CSF_	fM/h	1.75	29566794, 25720406
Brain ISF ptau production rate	K_in_ISF_	fM/h	7.06	Assumption
ptau half-life	—	Days	30.7	29566794
ptau elimination rate	K_out_	1/h	0.000941	29566794
Complex degradation rate	K_deg_	1/h	0.001	Assumption

### Tau physiologically-based pharmacokinetic-pharmacodynamic model validation in rats, monkey, and human

**FIGURE 2:**
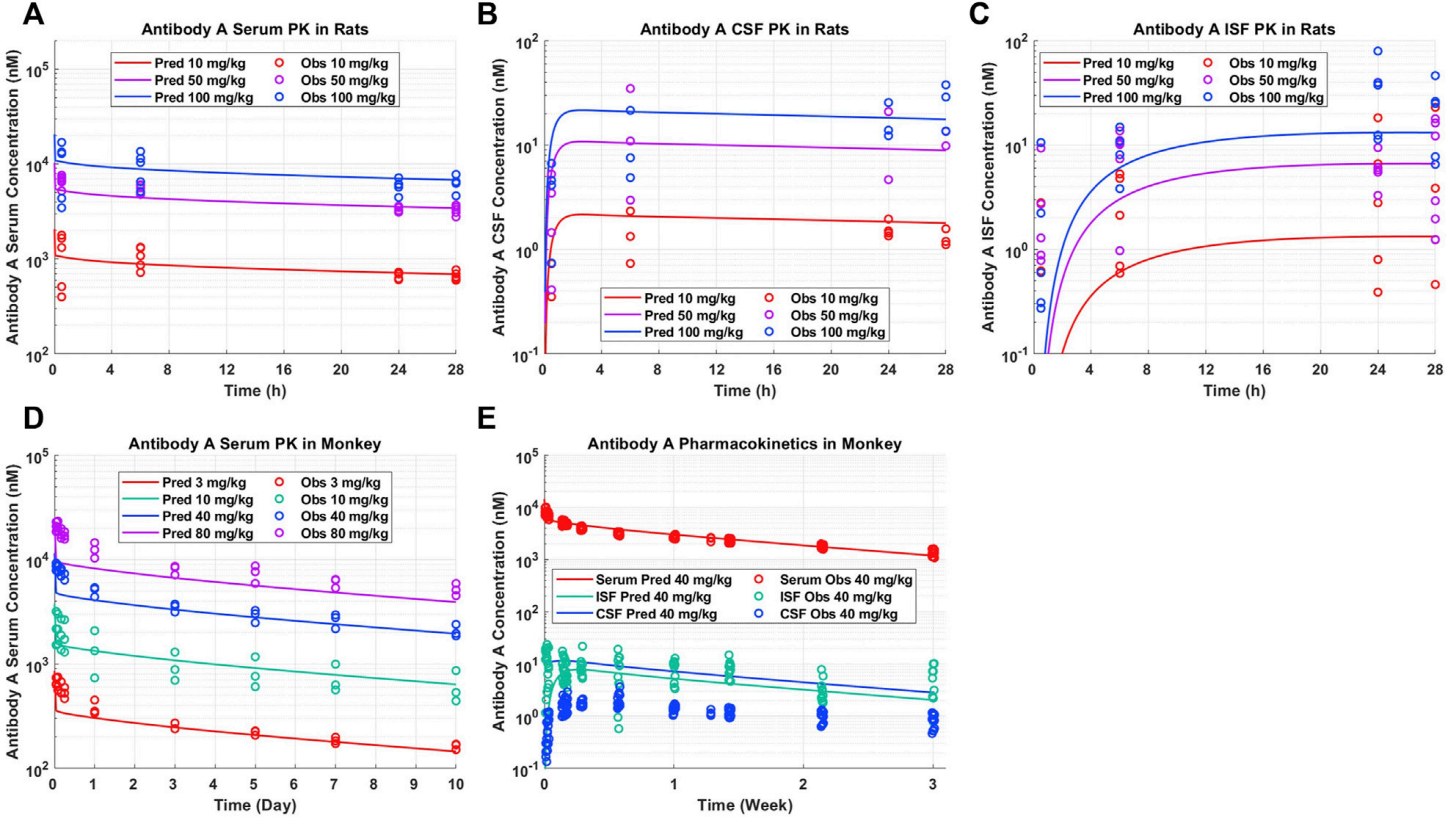
Antibody A pharmacokinetics in preclinical species. **(A)** Serum, **(B)** CSF, and **(C)** brain ISF concentrations in rats administered 10, 50, and 100 mg/kg IV. **(D)** Serum concentrations in cynomolgus monkeys administered 3, 10, 40, 80 mg/kg IV. **(E)** Serum, CSF, and brain ISF concentrations in rhesus monkeys administered 40 mg/kg IV. *A priori* model predictions are displayed as solid lines and observed data as circles.

*A priori* model simulations were able to adequately describe the pharmacokinetics of Antibody A in preclinical rat and NHP models in serum, CSF, and brain ISF across several dose levels (Figure 2). Antibody A is a humanized monoclonal antibody on an IgG4 backbone that specifically recognizes phosphorylated tau as assessed by ELISA, with a purity of 95% by SEC and SDS-PAGE. The serum concentrations of Antibody A in rats increased in a dose-dependent linear fashion, which was well captured by model predictions (Figure 2A). The CSF concentrations of Antibody A in rats increased in a dose-dependent linear fashion, paralleling serum concentrations, which was also well described by the model (Figure 2B). The brain ISF concentrations of Antibody A in rats increased in a dose-dependent linear fashion (Figure 2C). Model predictions were able to more accurately describe antibody concentrations at later time points and observations suggests that the distribution of the antibody in brain ISF occurs faster than what the model is currently predicting. However, there is a considerable amount of variability with this type of experiment.

The serum PK of Antibody A in cynomolgus monkeys increased in a dose-dependent linear fashion, which was well described by model predictions (Figure 2D). The serum and brain ISF concentrations of Antibody A in rhesus monkeys administered 40 mg/kg IV were well-predicted (Figure 2E). Unexpectedly, we observed a ∼10-fold lower exposure in CSF compared to brain ISF. The concentration of antibody in CSF was <0.05% of serum concentrations, which is less than the typical reported average of ∼0.1%–0.2% (Wang et al., 2018).

**FIGURE 3:**
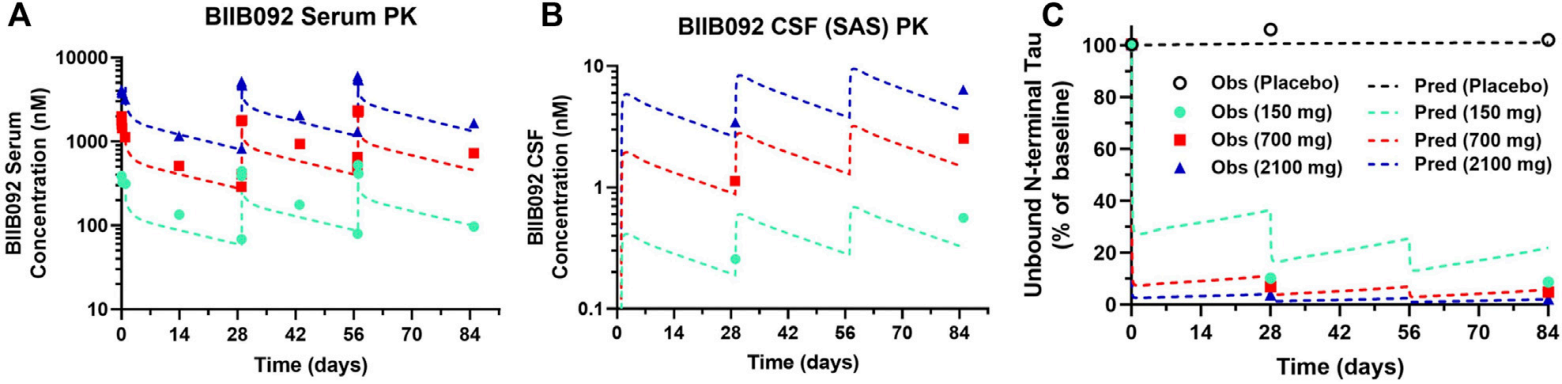
PBPK-PD model predictions overlayed with clinical BIIB092 pharmacokinetics and pharmacodynamics data. **(A)** Serum and **(B)** CSF concentrations of BIIB092 and **(C)** unbound N-terminal tau concentrations in CSF (relative to baseline). Three doses of BIIB092, 150, 700, and 2100 mg IV are depicted in cyan, red, and dark blue, respectively. Observed (Obs) data are represented as markers and model predictions (Pred) are represented by dashed lines.

Clinical PK-PD data for BIIB092, an anti-tau antibody, from a phase 1b study in progressive supranuclear palsy (PSP) patients was digitized from the literature (Boxer et al., 2019). Model predictions well captured observed BIIB092 serum (Figure 3A) and CSF (Figure 3B) concentrations. Model predictions well described the decrease in unbound N-terminal tau for the medium (700 mg) and high (2100 mg) doses of BIIB092, but slightly underpredicted the level of target engagement for the lowest dose (150 mg) (Figure 3C).

### Tau antibody affinity optimization and half-life extension simulations

**FIGURE 4:**
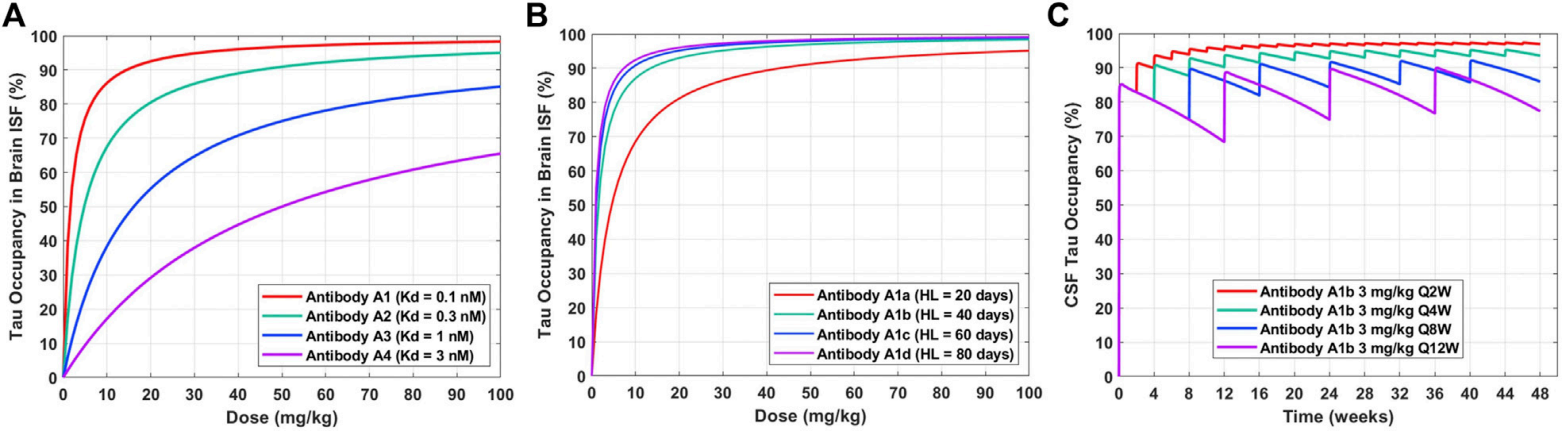
Model predictions for the impact of antibody affinity, half-life, and dosing frequency on tau dynamics in humans after a single IV dose. **(A)** Tau occupancy in brain ISF at 8 weeks as a function of dose for four theoretical antibodies (A1–A4) with varying affinities (0.1–3 nM). **(B)** Tau occupancy in brain ISF at 8 weeks as a function of dose for four theoretical antibodies (A1a–A1d) with the same affinity (Kd = 0.1 nM) and varying elimination half-lives (20–80 days). **(C)** CSF tau occupancy as a function of time for Antibody A1b (Kd = 0.1 nM, HL = 40 days) administered at a dose of 3 mg/kg IV for four different dosing frequencies (Q2W, Q4W, Q8W, Q12W).

The validated PBPK-PD model can be applied to understand the impact of anti-tau antibody affinity optimization and half-life extension on dose regimen. The tau occupancy in brain ISF at 8 weeks after a single IV dose for four theoretical antibodies (A1, A2, A3, A4) with different binding affinities to tau (0.1, 0.3, 1, 3 nM) was simulated across a range of doses (0–100 mg/kg) (Figure 4A). This enabled a quantitative understanding of the impact of antibody affinity on the dose required to effectively engage tau in human brain ISF. We observed that a dose of approximately 15 mg/kg and antibody affinity of 0.1 nM would be required to achieve 90% target occupancy at 2 months post administration (Figure 4A, Antibody A1). An antibody with an affinity of 0.3 nM would require a dose of 50 mg/kg to achieve the same level of occupancy (Figure 4A, Antibody A2). Antibodies with an affinity greater than 1 nM would likely not be clinically viable, due to very large required doses >100 mg/kg for a dosing frequency of every 8 weeks (Figure 4A, Antibody A3 and A4).

Tau occupancy in brain ISF at 8 weeks after a single IV dose for four antibodies (A1a, A1b, A1c, A1d) with the same binding affinity to tau (0.1 nM) and different elimination half-lives (20, 40, 60, 80 days) was simulated across a range of doses (0–100 mg/kg) (Figure 4B). This enabled a quantitative understanding for the impact of half-life on the dose required to effectively engage tau in human brain ISF. We observed that improvements in antibody half-life could significantly reduce the dose required to obtain high levels of tau occupancy in brain ISF (e.g. >90%). For example, a 3-fold increase in half-life (Antibody A1a vs. A1c; 20 vs. 60 days) resulted in a 4.5-fold reduction in the dose required to achieve 90% tau occupancy (45 mg/kg vs. 10 mg/kg). Additionally, our predictions suggest that there is not much of an added benefit between a half-life of 60 days compared to 80 days (Antibody A1c vs. A1d).

To understand how different clinical dosing regimens would impact tau engagement in the CSF, we performed simulations for an anti-tau antibody (Antibody A1b) with an affinity of 0.1 nM and half-life of 40 days (Figure 4C). We predicted the occupancy of tau over 48 weeks in human CSF for 3 mg/kg of Antibody A1b administered IV at four different dosing frequencies every 2 (Q2W), 4 (Q4W), 8 (Q8W), and 12 (Q12W) weeks. Model predictions suggest that a dosing regimen of 3 mg/kg Q4W will achieve >90% CSF tau occupancy after the 3^rd^ dose (week 8) and at steady-state. However, less frequent administrations, Q8W and Q12W, were unable to achieve high levels of target engagement (>90%) in the brain. Simulations of this nature have been valuable for anticipating the level of tau engagement at the site of action for various clinical dosing regimens of interest.

### Predictions for the impact of blood contamination on cerebral spinal fluid samples

**FIGURE 5:**
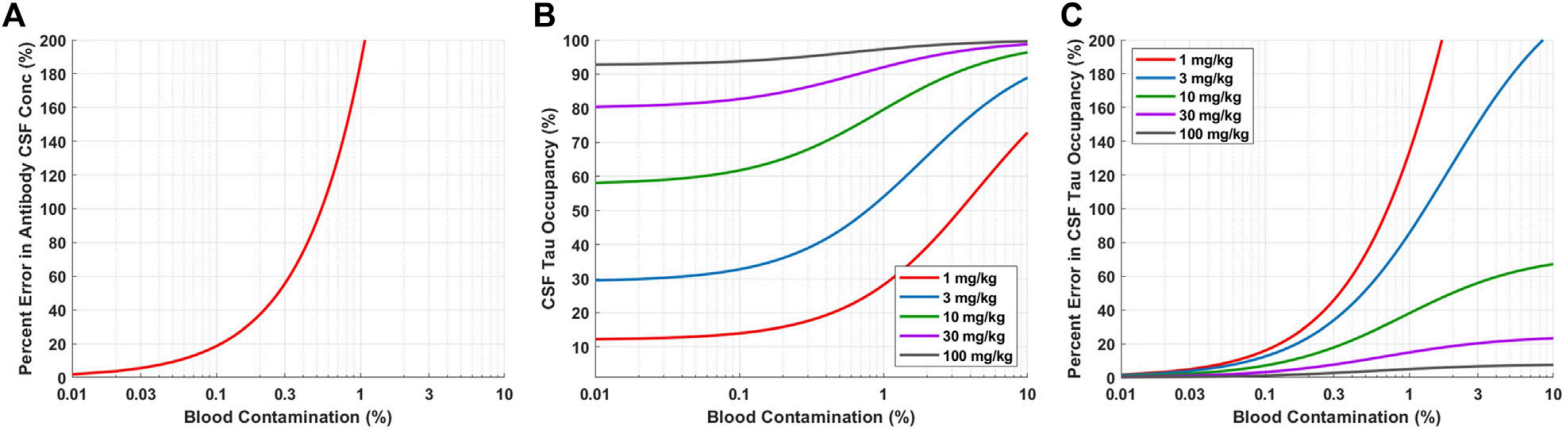
Model predictions for impact of blood contamination on antibody concentration and tau occupancy in CSF. **(A)** Predicted percent error in CSF concentration of Antibody A for various levels of blood contamination. **(B)** Predicted CSF tau occupancy for various doses and levels of blood contamination. **(B)** Predicted percent error in CSF tau occupancy for various doses and levels of blood contamination.

For neuroscience therapeutics, CSF concentrations are often used as a surrogate for concentrations at the site of action (brain ISF). CSF is typically collected in the clinic via lumbar punctures. However, there is concern of potential blood contamination with this collection methodology. We utilized a quantitative approach to assess the impact of blood contamination on antibody concentrations and tau dynamics in CSF samples (Figure 5). CSF concentrations of Antibody A were calculated for a range of different levels of blood contamination using Eq. 4. Blood contamination in CSF of 0.1, 0.6, and 1.0% results in a percent error in antibody CSF concentrations of 20, 100, and 200%, respectively (Figure 5A). Blood contamination appears to start impacting CSF tau occupancy around 0.1% (Figure 5B). However, the impact on in CSF tau occupancy is dependent upon the dose administered. The percent error in CSF tau occupancy exemplifies this phenomenon, where low doses are more significantly impacted by blood contamination compared to higher doses (Figure 5C). Since CSF antibody concentrations for high doses are already close to saturating target binding, additional antibody exposure from blood does not meaningfully increase target occupancy.

### Clinical trial predictions for tau antibody pharmacokinetic-pharmacodynamic

**FIGURE 6:**
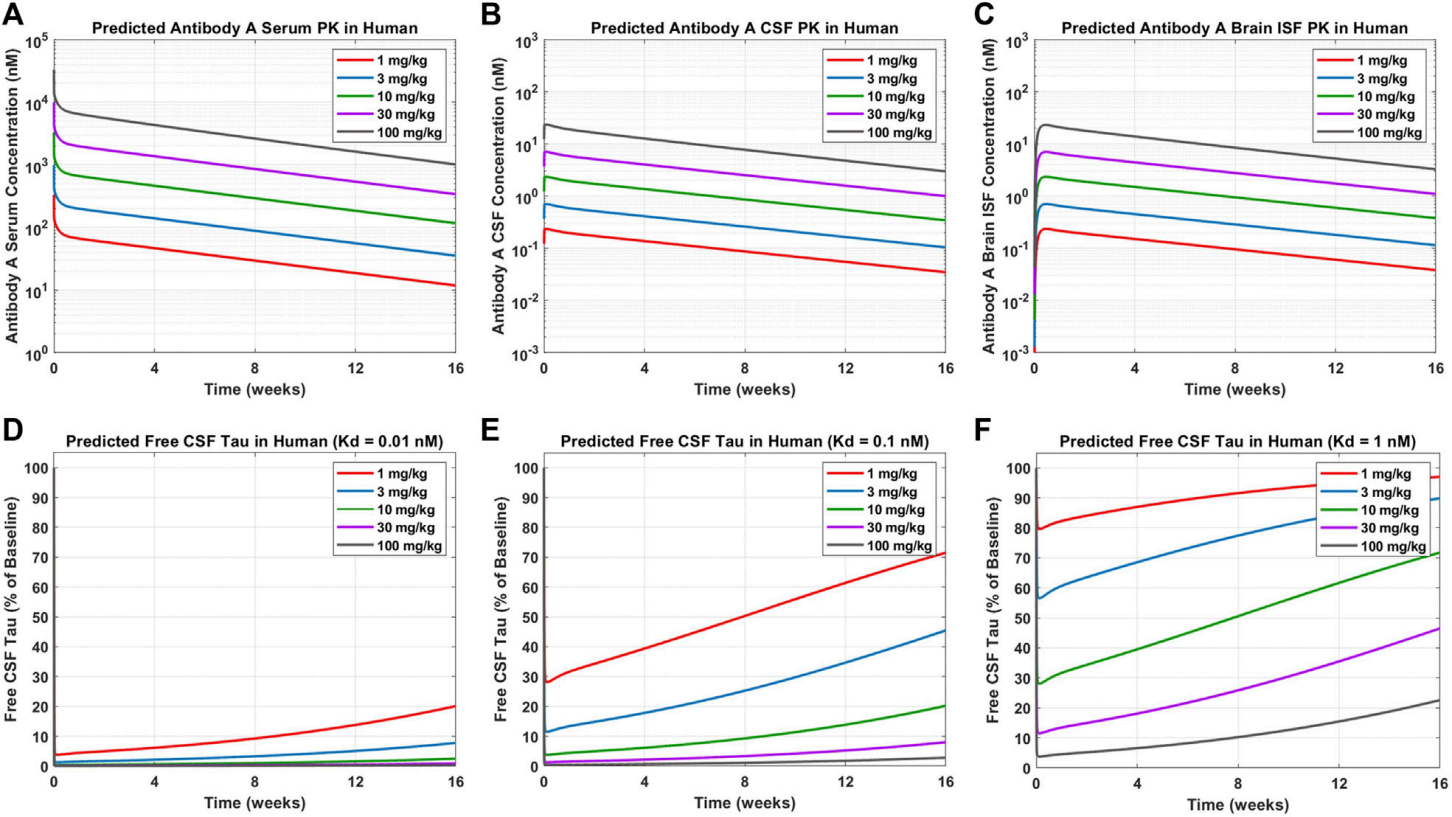
Model predictions for Antibody A PK and CSF ptau dynamics in humans. Antibody A concentrations in human **(A)** serum, **(B)** CSF, and **(C)** brain ISF at a single IV dose of 1, 3, 10, 30, 100 mg/kg. The change in free CSF tau is shown for three different scenarios of different antibody affinities to tau: **(D)** 0.01 nM, **(E)** 0.1 nM, and **(F)** 1 nM. Antibody and antibody-target complex elimination half-life was assumed to be approximately 40 days.

Simulations were performed to predict Antibody A concentrations in serum, CSF, and brain ISF as well as tau dynamics in CSF in humans administered a single IV dose of 1, 3, 10, 30, and 100 mg/kg (Figure 6). Antibody A concentration in the brain is approximately three orders of magnitude less than antibody concentrations in serum (Figures 6A–C). The CSF-to-serum ratio for antibody concentration in the model is ∼0.3%, which is in agreement with previously reported clinical observations (Jankovic et al., 2018). Free CSF tau dynamics, depicted as percentage from baseline, were predicted for antibodies with three different affinities (0.01, 0.1, and 1 nM). The antibody with high affinity (0.01 nM), free CSF tau was reduced below 10% across all dose levels for at least 1 month (Figure 6D). The antibody with medium affinity (0.1 nM), free CSF tau was reduced below 10% for three of the dose groups (10, 30, and 100 mg/kg) for at least 1 month (Figure 6E). The antibody with low affinity (1 nM), free CSF tau was reduced below 10% only in the highest dose group (100 mg/kg) for at least 1 month (Figure 6F). These simulations help to inform what doses to select to achieve a desired response and to ensure the characterization of the full pharmacodynamic profile.

## Discussion

We have demonstrated the application of PBPK-PD modeling to support various aspects of preclinical and early clinical development of antibody therapeutics for the treatment of Alzheimer’s disease and tauopathies. The model was validated using preclinical and clinical data for antibody pharmacokinetics in serum, CSF, and brain ISF as well as clinical data for tau engagement in CSF. *A priori* predictions of antibody PK and tau dynamics in human serum and brain ISF/CSF were useful for understanding relationships between antibody affinity/half-life and target engagement, which may inform first-in-human dose selection and design of phase I clinical trials. We have only reported mean predictions as the level of variability around parameter values is unknown. Clinical population PK-PD data would help to inform inter-individual variability. The mechanistic PBPK approach offers an alternative to allometry for predicting serum exposures, but it’s unclear whether a PBPK approach would be more or less predictive than traditional allometric scaling for preclinical-to-clinical translation of drug exposures. A head-to-head comparison between the two different approaches across multiple antibody therapeutics would be valuable.

Blood contamination may occur when collecting CSF via lumbar puncture. Blood contamination could change the concentration of drug and target in the CSF samples, which ultimately may alter the interpretation of target engagement. Therefore, it is crucial to quantitatively understand the level of blood contamination in each sample and perform an adjustment for the concentration of drug in CSF accordingly (Eq. 4). Simulations are able to help guide the selection of a threshold for an acceptable level of blood contamination where adjustments to the concentration of drug in CSF may not be required. For example, setting the acceptable level of blood contamination to <0.1% ensures that the percent error for the concentration of drug in CSF is <20% (Figure 5A). Blood contamination in CSF can determined by the concentration of hemoglobin in CSF relative to serum.

Simplifying assumptions were made throughout the model development process. We describe tau using a single compartment. However, pathological tau is present intra- and extra-cellularly, undergoes complex aggregation processes and post-translational modifications, and spreads throughout the brain in a transneuronal fashion. We have assumed one-to-one binding, where an antibody is able to only bind to a single target. In reality, a single antibody could bind to two targets as well as multiple antibodies could bind to one oligomeric protein aggregate. We used the molecular weight of monomeric protein for conversions to molar units. Although this approach accounts for multiple antibodies binding to oligomeric tau, it makes the assumption that antibodies are able to bind to all tau monomers within an oligomer. This may not be appropriate as tau proteins within an oligomer could create steric hinderance by shielding the antibody binding site of other tau proteins. However, from a modeling perspective, its not entirely clear on how the molecular weight for aggregated proteins with multiple binding sites should be best considered, especially when working with protein aggregates of variable sizes. We assumed static target-mediated drug disposition (TMDD), where TMDD processes occur independently in each brain compartment. In other words, there is no distribution of target or antibody-target complex between brain compartments. A dynamic TMDD model would include additional kinetic processes, such as endosomal uptake/escape, FcRn binding, and paracellular transport of the target and antibody-target complex.

The publicly available clinical data assessing the engagement of tau-antibodies in human CSF is currently only in PSP patients (Boxer et al., 2019). Considering the differences in tau biology between PSP and AD, data from AD patients is required for further model validation. The current parameterization of the model (tau abundance and turnover) represents a cognitively normal to early AD population. The change in free tau was not sensitive to changes in tau concentration and turnover within patho-physiologically reported ranges, which may be reflective of the very low concentrations of target (∼ pM). However, different parameterizations of the model should be made depending upon the patient population and tau therapy of interest, which could improve the accuracy of predictions across different stages of Alzheimer’s disease. Sato et al. (2018) is the only paper to our knowledge that has measured the turnover rate of tau in humans (∼30 days). There are additional papers that have measured CSF tau concentrations in Alzheimer’s disease. Andreasen et al. (1998) reported CSF tau concentrations in AD patients, which ranged from 5 to 33 p.m.. Riemenschneider et al. investigated CSF tau concentrations in AD and mild cognitive impairment (MCI) subjects, which ranged from ∼2 p.m. in age-matched controls to ∼10 p.m. in AD/MCI subjects (Riemenschneider et al., 2002). Overall, CSF tau concentrations across healthy elderly and AD patients appear to range between single to double digit picomolar and are comparable to the CSF tau concentration used in Table 1 (27 p.m.). The concentration of tau in brain ISF is more challenging to obtain as this value is typically obtained through brain microdialysis studies. Relationships could be explored between predicted changes in free brain ISF tau and data from longitudinal tau PET imaging studies.

As more data begins to emerge from clinical trials investigating tau-targeted therapies in AD patients (Congdon and Sigurdsson, 2018; Ayalon et al., 2021), a QSP model platform could be developed for tau pathology in AD or an existing model could be expanded. For example, Madrasi et al. in 2020 developed an AD QSP model for amyloid-targeted therapies (Madrasi et al., 2021), which could be expanded to include tau biology. There are many complexities of tau biology that could be considered in future model development, such as detail on tau aggregation kinetics, tau isoforms and post-translational modifications, spatial localization, and various routes of tau spreading. Some of these features have been included in QSP models of tau pathology (Karelina et al., 2021). Systems models could include FcγR and clearance through microglial phagocytosis, depending on the effector function status of tau antibodies, as well as other neuroimmunological components. Data for tau peptide concentrations in clinical CSF samples from patients with neurodegenerative disease could be used to understand engagement toward different tau peptides and isoforms (Barthelemy et al., 2016). However, more detail on the exact concentration of tau fragments would be required. Tau seeding and spreading kinetics has been shown to be dependent upon differences in tau protein conformation and post-translational modifications, such as high molecular weight forms of soluble tau and the extent/site of phosphorylation (Dujardin et al., 2020). Differences in tau seeding propensity has been able to partially explain inter-individual differences in the rate of clinical neurodegenerative disease progression (Dujardin et al., 2020). Developing a mechanistic quantitative model that captures the complexities of tau pathology could help towards understanding clinical heterogeneity in disease progression and treatment response.

## Conclusion

Our work exemplifies the utility of PBPK-PD modeling to address challenges faced in preclinical development and clinical translation of anti-tau antibody therapeutics for the treatment of Alzheimer’s disease. This modeling approach provides a foundation that can be further expanded to incorporate additional complexities of tau biology. This tau PBPK-PD model can also be refined as clinical data emerges to inform late stages of clinical development. However, the size of this platform model may limit its applicability. Minimal PBPK models of the brain provide a framework that can be more easily adapted to incorporate targets of interest and integrated with quantitative systems pharmacology models of neurological diseases.

## Data Availability

The raw data supporting the conclusions of this article will be made available by the authors, without undue reservation.
